# Predictivity of clinical, laboratory and imaging findings in diagnostic definition of palpable thyroid nodules. A multicenter prospective study

**DOI:** 10.1007/s12020-018-1577-5

**Published:** 2018-03-22

**Authors:** Maria Grazia Chiofalo, Simona Signoriello, Franco Fulciniti, Nicola Avenia, Serenella Ristagno, Celestino Pio Lombardi, Angelo Nicolosi, Maria Rosa Pelizzo, Giuliano Perigli, Andrea Polistena, Vincenzo Panebianco, Rocco Bellantone, Pietro Giorgio Calò, Isabella Merante Boschin, Benedetta Badii, Massimo Di Maio, Ciro Gallo, Francesco Perrone, Luciano Pezzullo

**Affiliations:** 1 Istituto Nazionale per lo studio e la Cura dei Tumori, Fondazione G. Pascale, IRCCS, Napoli, Italy; 2 Dipartimento di Salute Mentale e Fisica e Medicina Preventiva, Università della Campania Luigi Vanvitelli, Napoli, Italy; 3 Endocrinochirurgia, Università di Perugia, Terni, Italy; 4 Endocrinochirurgia, Ospedale di Taormina, Taormina, Italy; 5 Chirurgia Endocrina, Policlinico Gemelli, Università Cattolica del Sacro Cuore di Roma, Rome, Italy; 6 Dipartimento di Scienze Chirurgiche, Università di Cagliari, Cagliari, Italy; 7 Patologia Speciale Chirurgica, Azienda Ospedaliera Universitaria di Padova, Padova, Italy; 8 Endocrinochirurgia e Chirurgia Miniinvasiva, Azienda Ospedaliera Universitaria Careggi, Firenze, Italy; 9 Present Address: Istituto Cantonale di Patologia, Locarno, Switzerland; 10 Present Address: Università di Torino, Turin, Italy

**Keywords:** Thyroid nodules, Cytology, Prospective observational trial, Multiple correspondence analysis, Diagnostic accuracy

## Abstract

****Purpose**:**

To assess the role of clinical, biochemical, and morphological parameters, as added to cytology, for improving pre-surgical diagnosis of palpable thyroid nodules.

****Methods**:**

Patients with a palpable thyroid nodule were eligible if surgical intervention was indicated after a positive or suspicious for malignancy FNAC (TIR 4–5 according to the 2007 Italian SIAPEC-IAP classification), or two inconclusive FNAC at a ≥3 months interval, or a negative FNAC associated with one or more risk factor. Reference standard was histological malignancy diagnosis. Likelihood ratios of malignancy, sensitivity, specificity, negative (NPV), and positive predictive value (PPV) were described. Multiple correspondence analysis (MCA) and logistic regression were applied.

****Results**:**

Cancer was found in 433/902 (48%) patients. Considering TIR4–5 only as positive cytology, specificity, and PPV were high (94 and 91%) but sensitivity and NPV were low (61 and 72%); conversely, including TIR3 among positive, sensitivity and NPV were higher (88 and 82%) while specificity and PPV decreased (52 and 63%). Ultrasonographic size ≥3 cm was independently associated with benignity among TIR2 cases (OR of malignancy 0.37, 95% CI 0.18–0.78). In TIR3 cases the hard consistency of small nodules was associated with malignity (OR: 3.51, 95% CI 1.84–6.70, *p* < 0.001), while size alone, irrespective of consistency, was not diagnostically informative. No other significant association was found in TIR2 and TIR3.

****Conclusions**:**

The combination of cytology with clinical and ultrasonographic parameters may improve diagnostic definition of palpable thyroid nodules. However, the need for innovative diagnostic tools is still high.

## Introduction

The discovery rate of thyroid nodules has widely risen in last decades, mainly due to the detection of clinically unapparent lesions by means of ultrasonography (US) [1]. This clearly emerged by an old prospective study comparing palpation and US in a population of subjects without known thyroid disease [2]. Authors reported an US prevalence as high as 67%, whereas palpable nodules were detected in only 21% of the cohort. However, the actual clinical utility of discovering such a quantity of non-palpable nodules seems to be poor as the risk of malignancy is negligible [3]. Palpable thyroid nodules are reported in approximately 4–7% of the population [4, 5]. In this setting, the rate of malignancy is more relevant, being estimated from 8 to 16% [6, 7]. Therefore, palpable thyroid lesions may be considered as a separate clinical entity requiring a specific diagnostic approach.

To date, several guidelines on the management of thyroid nodules have been published, but a clear distinction between palpable and non palpable lesions has never been performed [6, 8–14]. Furthermore, a number of clinically relevant topics are still debated, and need to be addressed by additional studies. The areas of uncertainty concerns, for instance, the use of ultrasound scan criteria to assess cancer risk, the indication for fine-needle aspiration cytology (FNAC), the new diagnostic and therapeutic techniques, including molecular markers, and the optimal follow-up strategy for patients with benign thyroid nodules.

The evaluation of thyroid nodules includes clinical examination, ultrasound, fine needle aspiration cytology. Currently, FNAC is considered the most accurate test, for evaluation of thyroid nodule due to the high sensitivity (83%) and specificity (92%) [15, 16].

The available literature about the diagnostic accuracy of FNAC, however, is based almost exclusively on retrospective studies performed in cases of positive or indeterminate cytology. Currently, only patients with positive FNAC undergo surgery, thus, for the majority of cases with negative cytology, a histological correlation is not available. The question of false negatives is addressed in several studies [8, 17–23]. A rate of 25% of false negatives has been reported. Importantly, this percentage is even higher in large palpable nodule (≥4 cm) [17, 24]. This suggests that FNAC sensitivity may be even more limited in the setting of palpable nodules, where up to a third of cases of malignancy may be missed [22].

Aim of the present study is to assess the role of clinical, biochemical, and morphological parameters as auxiliary tools, added to cytology, for improving pre-surgical diagnosis of palpable thyroid nodules.

## Materials and methods

### Study design

We performed a multicentre prospective observational study to describe the predictive value for diagnosis of malignancy of several clinical, laboratory and radiological parameters, commonly measured in routine clinical practice. The study protocol was approved by the ethical committees at all participating Institutions. Patients were consecutively enrolled at seven participating centers.

### Study population

Patients ≥18 years old were eligible if they had a palpable thyroid nodule, for which surgical intervention was indicated. In details, surgery was indicated when at least one of the following three conditions was present: (1) cytological examination positive or suspicious of malignancy (TIR 4-5 according to the 2007 Italian SIAPEC-IAP classification [25]), (2) two inconclusive cytological examinations with a ≥3 months interval, (3) cytological examination negative for malignant cells (TIR 2-3) associated with at least one major and two minor risk criteria. Major and minor risk criteria were arbitrarily defined by the study protocol. Major risk criteria included clinical (previous neck irradiation), biochemical (calcitonin levels more than twice the upper normal value in absence of renal failure and of pump inhibitors use or positivity at the pentagastrin stimulation test) or ultrasonographic (hypoechogenicity, irregular margins, major diameter ≥3 cm, microcalcifications). Minor risk criteria included clinical (hard consistency of the nodule, age >45, male gender), biochemical (human thyreoglobulin [HTG] > 2000 ng/ml) or ultrasonographic (increase of the major diameter in patients on treatment with thyroxine ≥5 mm if previous diameter was <1 cm or ≥1 cm if previous diameter was ≥1 cm, intranodular vascularization at Doppler examination).

Patients were excluded in case of concomitant diseases preventing surgery, evidence of locally advanced disease or metastases (lymph nodes or other distant sites). Also patients with non-palpable nodes were excluded. Informed consent was obtained from all individual participants included in the study.

### Study procedures

Eligible patients underwent FNAC and surgery, independently of the result of cytology. Ultrasonographic evaluation was performed according to participating center practice by experienced radiologists and no independent confirmation was mandated by the protocol.

Cytology interpretation was based on the 2007 Italian SIAPEC-IAP 5-category scale: TIR1: non-diagnostic, inadequate (smears should contain 6 or more groups of at least 10 thyroid follicular cells to be considered adequate); TIR2: benign (consistent with nodular goiter or thyroiditis); TIR3: indeterminate; TIR4: suspicious of malignancy; TIR5: malignant [25]. Palpable nodules >2 cm or containing microcalcifications had to be sampled with at least two needle passes. Samples with macroscopic blood contamination had to be repeated immediately, when feasible [26, 27]. There was no independent revision of cytological and histological diagnosis, according to a pragmatic approach, considering that all participating centers are considered as referral Institutions for thyroid cancer.

### Statistical analysis

Diagnostic performance of FNAC was assessed using likelihood ratio (LR), sensitivity, specificity, negative predictive value (NPV), and positive predictive value (PPV); for all the estimated 95% confidence intervals were calculated. Reference standard was histological diagnosis (malignant/benign). Three categories of FNAC were used (TIR2, TIR3, and TIR4-5). LR was estimated for each FNAC category. Since the other measures of accuracy (sensitivity, specificity, PPV and NPV) can only be calculated with dichotomous tests, we estimated them twice, considering as positive in turn either TIR4 and TIR5 categories (TIR4-5) or the previous ones combined with the TIR3 category (TIR3-4-5).

Associations of clinical, biochemical and ultrasonographic variables with malignancy were separately evaluated in patients with TIR2 and TIR3 categories. Surgery indeed was mandatory in patients with TIR4 or TIR5 cytological diagnosis and any further information would be irrelevant for clinical decision.

Univariate associations were tested using Pearson’s chi-square test and Fisher’s exact test, as appropriate. Multivariable analyses were performed by means of multiple logistic regression models.

Since the six ultrasonographic variables were expected to be highly correlated, relationships between them were preliminary investigated with multiple correspondence analysis (MCA) [28]. MCA is a graphical technique that allows to display categories of variables as points in a geometric map of relationship. The plot axes indicate latent variables that are derived from the contribution of the single variables. Proximities of categories in the two-dimensional plot suggest possible associations between them. As a general rule the distance between two points may be roughly interpreted as the correlation among them although findings must also be read in the light of the quantitative contributions of the categories to the axes (*η*^2^). The percentage of information explained (i.e., the percentage of total variability explained by the two axes of the plot) was measured following Benzécri: higher the percentage better the two-dimensional representation of data [29]. To improve the interpretation of results, the two histology categories (malignant/benign) were depicted on the MCA plot as supplementary variables (i.e., they did not contribute to the axes). As a result of the exploratory MCA the six ultrasonographic variables were combined in two new variables that were entered as covariates in the logistic models.

For all analyses, results were considered statistically significant if the two-tailed *p*-value was 0.05 or less. All analyses were performed with R software, version 3.0.0 [The R Foundation for Statistical Computing 2013].

## Results

Overall 914 eligible patients were enrolled at seven centers between March 2010 and April 2012. Twelve patients underwent surgery because of two consecutive inconclusive cytological examinations; these patients were excluded from subsequent analysis. Baseline characteristics of the remaining 902 patients are reported in Table 1. Patients were prevalently females (76%) with a median age of 49 years. Biochemical variables were found positive in a very small number of patients (23 cases overall). FNAC categories were evenly distributed in the study patients (33% TIR2, 35% TIR3 and 32% TIR4-5). A malignant tumor was present in 48% of patient, mostly as papillary carcinoma (86%).

**Table 1:** Baseline characteristics of the study patients

Variable	*N* = 902
Age at diagnosis years, median (range)	49 (18–87)
Clinical variables	
Previous neck irradiation	5 (1%)
Hard consistency at palpation	428 (47%)
Age > 45 years	553 (61%)
Male gender	213 (24%)
Biochemical variables	
Serum calcitonin level > 2 UNL	12 (1%)
Positive pentagastrin stimulation test	3 (0%)
Serum thyreoglobulin > 2000 ng/ml	8 (1%)
Ultrasonography variables	
Hypoechogenicity	719 (80%)
Irregular margins	348 (39%)
Size ≥ 3 cm	428 (47%)
Microcalcifications	379 (42%)
Increase in nodule volume	216 (24%)
Intranodular vascularization	624 (69%)
Fine-needle aspiration category	
TIR2	295 (33%)
TIR3	315 (35%)
TIR4-5	292 (32%)
Malignant histology	435 (48%)
Papillary carcinoma	374 (41%)
Follicular carcinoma	46 (5%)
Anaplastic carcinoma	5 (1%)
Medullary carcinoma	8 (1%)
Missing	2 (0%)
City of the participating center	
Terni	210 (23%)
Taormina	196 (22%)
Roma	124 (14%)
Napoli	114 (13%)
Cagliari	108 (12%)
Padova	81 (9%)
Firenze	69 (8%)

Estimated LRs for each TIR category are reported in Table 2. As expected the two extreme categories (TIR4-5 and TIR2) provided stronger information for or against malignancy, respectively, while the intermediate category (TIR3) was less informative. To estimate the other measures of accuracy (Table 3) two 2 × 2 contingency tables were derived where the intermediate category TIR3 was alternatively considered negative (and pooled with TIR2) or positive (and pooled with TIR4-5). When a “positive” result was limited to TIR4-5 category, sensitivity was equal to 61% (i.e., there was a false negative rate of 39%) and specificity was equal to 94%, meaning that only 6% of false positive patients were found. Accordingly PPV was high. Conversely, when the indeterminate TIR3 category was combined with TIR4-5 in the definition of ‘positive’ result, sensitivity increased to 88% (i.e., 12% of patients with malignancy were found negative), but specificity decreased to an unacceptably value of 52%, meaning that 48% patients without malignancy were found positive. Accordingly PPV value was only 63%.

**Table 2:** Diagnostic accuracy of fine-needle aspiration (FNA) category

	Histology				
FNA category	Malignant	Benign	Total	LR	(CI 95%)
TIR2	53	242	295	0.23	(0.18–0.31)
TIR3	116	199	315	0.63	(0.52–0.76)
TIR4-5	266	26	292	10.98	(7.5–16.08)
Total	435	467	902		

Likelihood ratios for malignancy

**Table 3:** Sensitivity, specificity and predictive values for malignancy (95% confidence intervals)

Definition of test outcome	Sensitivity	Specificity	PPV	NPV
Positive: TIR4-5 Negative: TIR2-3	61% (56–66%)	94% (92–96%)	91% (87–94%)	72% (69–76%)
Positive: TIR3-4-5 Negative: TIR2	88% (84–91%)	52% (47–56%)	63% (59–67%)	82% (77–86%)

*PPV* positive predictive value, *NPV* negative predictive value

Results of univariate analyses are summarized in the supplementary Table online separately for FNAC negative (TIR2) and indeterminate (TIR3) subjects. In the TIR2 subjects, size ≥ 3 cm was significantly associated with the absence of malignancy while no statistically significant association was found for any other variable. Within the TIR3 category, three variables were found to be associated with histology: hard consistency at palpation and irregular margins were more frequent in presence of malignancy while again size ≥ 3 cm was more frequently observed when malignancy was absent. Because of the very small prevalence, biochemical variables and previous neck irradiation were no further evaluated in multivariate analyses.

**Fig. 1:**
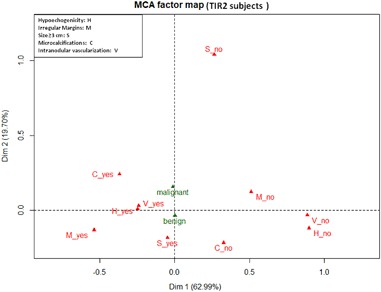
Multiple correspondence (MCA) two-dimensional plot in TIR2 patients

Exploratory MCA was performed separately in FNAC-TIR2 and FNAC-TIR3 patients. MCA two-dimensional plot in TIR2 patients is depicted in Fig. 1. The first two axes accounted for 83% of the total variability. The first axis could be mainly interpreted as expression of morphological modifications since it was associated with irregular margins, hypoechogenicity, intranodular vascularization and microcalcifications (*η*^2^ equal to 0.67, 0.52, 0.53, 0.29, respectively). The second axis was strongly associated only with size (*η*^2^ = 0.83). The histology categories, plotted as supplementary variables, were only slightly correlated with the second axis. Following this exploratory analysis, tumor size remained as single variable and a new ultrasonographic variable was derived, *morphological modifications*, summing up the categories on the same side along the first axis (presence of irregular margins, hypoechogenicity, intranodular vascularization, and microcalcifications). These two ultrasonographic variables were entered into the logistic model as covariates.

**Fig. 2:**
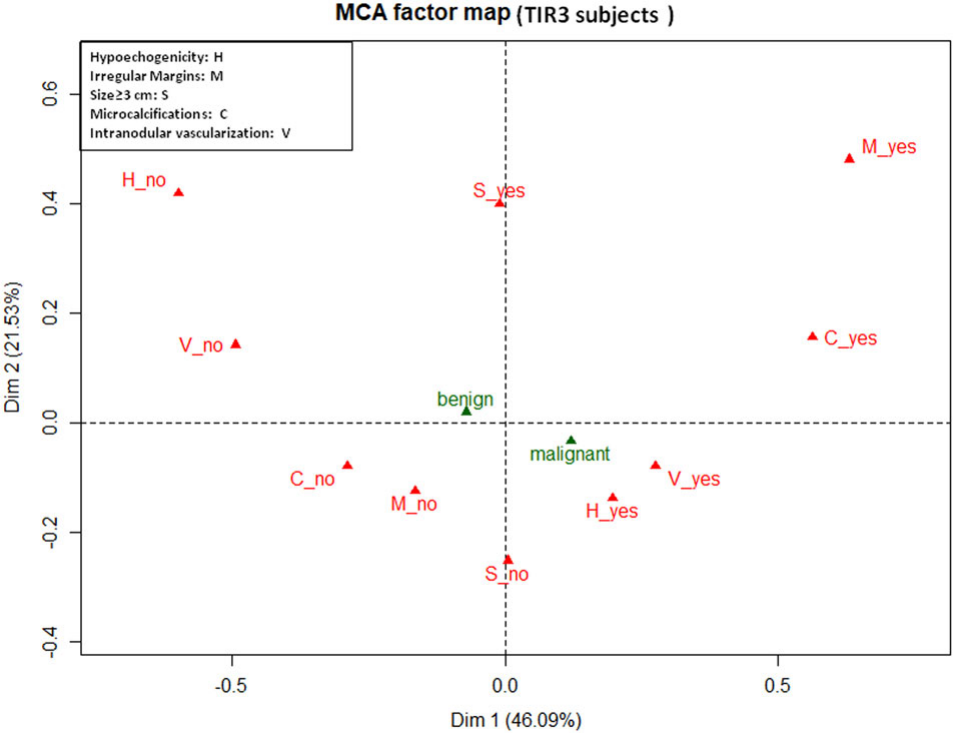
Multiple correspondence (MCA) two-dimensional plot in TIR3 patients

MCA two-dimensional plot in TIR3 patients is depicted in Fig. 2. The first two axes accounted for 68% of the total variability. The two axes may be read as above, and the first axis was associated with irregular margins, hypoechogenicity, intranodular vascularization and microcalcifications (*η*^2^ equal to 0.55, 0.38, 0.48, and 0.38, respectively). As above, size was associated with the second axis (*η*^2^ = 0.78). Following this exploratory analysis, the same two ultrasonographic variables were entered into the logistic model as covariates.

Results of the logistic regression analyses are reported in Table 4. The new variable “morphological modifications”, ranging from 0 to 4, was entered in the models as a continuous covariate. As for TIR2 cases, only size ≥ 3 cm was statistically associated with benign tumor (OR 0.37, 95% CI 0.18–0.78, *p* = 0.008). As for TIR3 cases, interaction between consistency and size was statistically significant (*p* = 0.028), therefore we entered into the model three dummy variables of combinations of consistency and size; soft consistency and size <3 cm was the reference category. It appeared that hard consistency combined with small size (<3 cm) was a strong predictor of malignancy (OR 3.51, 95% CI 1.84–6.70, *p* < 0.001), while the other two categories were not statistically significant. For a better understanding the distribution of malignant histology according to combination of size and consistency at palpation is reported in Table 5. The subgroup with the higher percentage of malignancy is the one with hard consistency at palpation and low nodule size.

**Table 4:** Logistic regression analysis

	OR	95% CI	*p*-value
TIR2			
Hard consistency at palpation	1.16	0.58–2.29	0.678
Age > 45 years	0.66	0.34–1.28	0.216
Male gender	1.55	0.76–3.16	0.224
Morphological modifications^a^	1.07	0.81–1.41	0.630
Size ≥ 3 vs. <3	0.37	0.18–0.78	**0.008**
TIR3			
Hard consistency and size <3 cm vs. soft consistency and size <3 cm	3.51	1.84–6.70	**<0.001**
Soft consistency and size ≥ 3 cm vs. soft consistency and size <3 cm	0.74	0.39–1.40	0.357
Hard consistency and size ≥ 3 cm vs. soft consistency and size <3 cm	0.79	0.35–1.76	0.563
Age > 45 years	0.91	0.55–1.51	0.719
Male gender	1.22	0.71–2.10	0.470
Morphological modifications^a^	1.26	0.99–1.59	0.056

^a^Sum of hypoechogenicity, irregular margins, microcalcifications, and intranodular vascularization (range 0–4)

bold significants to *p*-values

**Table 5:** Distribution of definitive histology by combination of consistency and size of the nodule in TIR3 cases

	Histology	
	Malignant	Benign
Hard consistency and size <3 cm	40 (68%)	19 (32%)
Hard consistency and size ≥3 cm	16 (33%)	29 (67%)
Soft consistency and size <3 cm	34 (31%)	77 (69%)
Soft consistency and size ≥3 cm	20 (27%)	55 (73%)
Total	116 (37%)	199 (63%)

## Discussion

To the best of our knowledge, this is the first study specifically focusing on palpable thyroid nodules, considered as a separate clinical entity, and trying to provide specific indications about the diagnostic work-up of this clinical setting.

A crucial feature of our paper is that the study population included patients with palpable thyroid nodules who meet surgical criteria, including both cytology results and clinical parameters considered as risk factors of malignancy. This explains why nearly a half of the study subjects, therefore much more than expected from a general cohort of patients with palpable nodules, were diagnosed with malignant disease at histology. By contrast, this also means that more than a half of patients advised to surgery basing on a pre-surgical cancer risk assessment actually harbor benign disease, thus indicating that current management of palpable thyroid nodules needs to be improved. Another relevant consequence of the composition of the cohort is that the study cannot provide information about FNAC accuracy, as taken alone. Indeed, only those subjects with benign cytology carrying clinical risk factors of malignancy have been included, and this represents a bias.

Therefore, the main aim of the study was to verify the diagnostic accuracy, as defined by the capability of identifying patients with cancer, of the combination between cytology and clinical parameters. Methodologically, a crucial point was the definition of ‘positive’ cytological result. Indeed, the TIR3 category is by definition a gray zone. Furthermore, the recently introduced TIR3 subcategories are not available as still not used in clinical practice at the time of the study, and this represents a limit of our analysis [30–32]. We therefore performed 2 separate analyses considering the TIR3 cytology as a negative (together with TIR2) and positive (together with TIR 4-5) result, respectively (Table 3.) When considering TIR 2-3 as the negative cytological result, the sensitivity was only 61%, which means that nearly 40% of malignant tumors were missed. By contrast, when TIR2 alone was considered as the only negative cytological category, the sensitivity rose to 88%, determining a negative predictive value of 82%. This means that the combination between TIR2 cytology and pre-surgical clinical assessment, as performed in this protocol, was related to a false negatives rate of only 12%, which was far more less than expected basing on literature data (as previously indicated).

In order to characterize specific diagnostic impact of the assessed clinical variables, we analyzed the relationship of each of them with final histology into the categories TIR2 and TIR3, as for TIR4-5 ones surgery is mandatory and any further information would be irrelevant for clinical decisions. Around 38% of cases categorized as TIR2 or TIR3 actually had cancer, consistent with some literature claims [22].

In both TIR2 and three categories, an independent relationship was found between large nodule size and benign histology. This implies that the likelihood of malignancy decreased in those palpable nodules with TIR2-3 cytology when the diameter is ≥3 cm. Therefore, basing on previous analysis, a TIR2 cytology from a large palpable nodule is related to low risk of false negativity. However, our finding that large tumors are associated with a lower risk of malignancy is not consistent with some reports that might be influenced by the fact only a subset of patients underwent preoperative FNAC [33, 34].

In the TIR3 category, hard consistency at palpation in small nodules was associated with malignancy and this may represent a useful indication for the management of palpable nodules with indeterminate cytology letting clinicians to choose thyroidectomy instead of follow-up.

The major strength of the present study is that the sample size is quite large, as derived from institutions with a high volume and high experience in the management of thyroid cancer, collected prospectively, and with definitive histological examination as gold standard for definition of performance of FNAC.

This study has, of course, also several limitations. As aforementioned, our population is not representative of the whole population of patients with palpable thyroid nodules, because only patients for whom surgery was indicated were included. As aforementioned, the recently introduced TIR3 subcategories were not used. Another issue is represented by the concordance between different pathologists [35]. Finally, inter-observer variability may also potentially affect ultrasonography [36].

In conclusion, the combination between cytology and assessment of clinical parameters (including clinics and US findings) represents a feasible diagnostic approach for palpable thyroid nodules. However, the need for innovative diagnostic tools for malignancy, driving decision making in clinical practice, is still high.

## Electronic supplementary material

Supplementary Information(DOCX 28 kb)
