# Supplementary material for: Predictivity of clinical, laboratory and imaging findings in diagnostic definition of palpable thyroid nodules. A multicenter prospective study
Source: Endocrine. 2018 Mar 22;61(1):43–50. doi: 10.1007/s12020-018-1577-5 (PMC5997121; doi:10.1007/s12020-018-1577-5)
Supplement: Supplementary file 1 — Supplementary Information(DOCX 28 kb) [file 12020_2018_1577_MOESM1_ESM.docx]

| Appendix Table A1. Association of clinical, biochimical and sonographic criteria with malignant histology, separately for TIR 2 and TIR 3 | | | | | | | | | |
| --- | --- | --- | --- | --- | --- | --- | --- | --- | --- |
|  | **TIR2** | | | |  | **TIR3** | | | |
| Risk factors | **Histological malignancy** | |  |  |  | **Histological malignancy** | |  |  |
|  | **yes**  **n=53** | **no**  **n=242** | **total**  **n=295** | **P*** |  | **yes**  **n=116** | **no**  **n=199** | **total**  **n=315** | **P*** |
| Clinical major  Previous neck irradiation  Clinical minor   - Hard consistency at palpation - Age >45 years - Male gender | 0 (0.0)  23 (43.4)  35 (66.0)  14 (26.4) | 1 (0.4)  94 (38.8)  180 (74.4)  51 (21.1) | 1 (0.3)  117 (39.7)  215 (72.9)  65 (22.0) | 0.64  0.54  0.22  0.40 |  | 1 (0.9)  56 (48.3)  71 (61.2)  35 (30.2) | 1 (0.5)  51 (25.6)  128 (64.3)  50 (25.1) | 2 (0.6)  107 (34.0)  199 (63.2)  85 (27.0) | 0.70  **<0.001**  0.58  0.33 |
| Biochemical major   - Serum calcitonine level > 2xUNL - Positive pentagastrin test   Biochemical minor   - Serum thyroglobulin > 2000 ng/ml | 0 (0.0)  0 (0.0)  2 (3.8) | 4 (1.7)  1 (0.4)  3 (1.2) | 4 (1.4)  1 (0.3)  5 (1.7) | 0.35  0.64  0.20 |  | 2 (1.7)  2 (1.7)  2 (1.7) | 1 (0.5)  0 (0.0)  0 (0.0) | 3 (1.0)  2 (0.6)  2 (0.6) | 0.28  0.06  0.06 |
| Sonographic major   - Hypoechogenicity - Irregular margins - Size ≥ 3 cm - Microcalcifications   Sonographic minor   - Intranodular vascularization | 43 (81.1)  24 (45.3)  39 (73.6)  28 (52.8)  43 (81.1) | 190 (78.5)  120 (49.6)  212 (87.6)  111 (45.9)  188 (77.7) | 233 (79.0)  144 (48.8)  251 (85.1)  139 (47.1)  231 (78.3) | 0.67  0.57  **0.009**  0.34  0.58 |  | 91 (78.4)  33 (28.4)  33 (28.4)  47 (40.5)  82 (70.7) | 146 (73.4)  32 (16.1)  89 (44.7)  60 (30.2)  120 (60.3) | 237 (75.2)  65 (20.6)  122 (38.7)  107 (34.0)  202 (64.1) | 0.31  **0.009**  **0.004**  0.06  0.06 |

*Chi-square or Fisher test, as appropriate; UNL=upper normal level
